# Augmented Reality, Serious Games and Picture Exchange Communication System for People with ASD: Systematic Literature Review and Future Directions

**DOI:** 10.3390/s22031250

**Published:** 2022-02-07

**Authors:** Haneen Almurashi, Rahma Bouaziz, Wallaa Alharthi, Mohammed Al-Sarem, Mohammed Hadwan, Slim Kammoun

**Affiliations:** 1 College of Computer Science and Engineering, Taibah University, Medina 42353, Saudi Arabia; haneenalmurashi1993@gmail.com (H.A.); rkammoun@taibahu.edu.sa (R.B.); msarem@taibahu.edu.sa (M.A.-S.); skammoun@taibahu.edu.sa (S.K.); 2 ReDCAD Laboratory, University of Sfax, Sfax 3029, Tunisia; 3 College of Community, Computer Sciences and Informatics, Taibah University, Medina 42353, Saudi Arabia; wharthi@taibahu.edu.sa; 4 Department of Computer Science, Saba’a Region University, Mareb 14400, Yemen; 5 Department of Information Technology, College of Computer, Qassim University, Buraydah 51452, Saudi Arabia; 6 Department of Computer Science, College of Applied Sciences, Taiz University, Taiz 6803, Yemen; 7 LaTICE Research Laboratory, University of Tunis, Tunis 1938, Tunisia

**Keywords:** Autism Spectrum Disorder, augmented reality, serious games, PECS’s application, learning environment

## Abstract

For people with Autism Spectrum Disorder (ASD), using technological tools, such as augmented reality (AR) and serious games remain a new and unexplored option. To attract people with ASD who have communicative, social, emotional and attention deficit disorders to behavioral treatments, an attractive environment is needed that ensures continuity during treatment. The aim of the current work is to efficiently examine systematic reviews and relevant primary studies on ASD solutions from 2015 to 2020, particularly those using the traditional Picture Exchange Communication System (PECS), the application of augmented reality and those that propose serious games, thereby providing an overview of existing evidence and to identify strategies for future research. Five databases were searched for keywords that may be included within the broad Autism Spectrum Disorder ‘ASD’ umbrella term, alongside ‘augmented reality’, ‘serious games’ and ‘PECS’. We screened 1799 titles and abstracts, read, and retained 12 reviews and 43 studies. The studies scrutinized in our systematic review were examined to answer four primary and four sub-research questions, which we formulated to better understand general trends in the use of approaches for attracting people with ASD to behavioral therapies. Additionally, our systematic review also presents ongoing issues in this area of research and suggests promising future research directions. Our review is useful to researchers in this field as it facilitates the comparison of existing studies with work currently being conducted, based on the availability of a wide range of studies in three different areas (AR, SG and PECS).

## 1. Introduction

Autism Spectrum Disorder (ASD) is a neurodevelopment condition that affects many abilities including social, verbal and physical skills. It typically arises in early childhood, before the age of three years [1]. The World Health Organization stated that, based on studies conducted in the past 50 years, it is expected that there will be an increase in the prevalence of ASD globally, with estimation that one in every 160 children will have an ASD. Researchers believe that there are three possible means for investigation the condition’s prevalence, i.e., by providing diagnostic tools, improvement in diagnostic criteria and improved awareness for autism spectrum disorders. Through diagnosis, the severity of autism spectrum disorders levels (low, medium, high) based on individual verbal intelligence quotient and the degree of language delay [2], which can evaluate the extent to which daily life is negatively affected. In some instances involving those with ASD, individuals may appear ‘normal’ to a stranger; however, once they interact with the ASD individual, they may begin to observe differences [1]. Many who have ASD are shy in terms of communicating or panic when the need arises to have a simple conversation. As such from poor communication, it can be difficult for the person with ASD to be left alone because it will negatively affect society. Since those with ASD are part of society, they also need to communicate and express their feelings. If a person with ASD receives treatment intervention early on in childhood, they will be more likely to lead an independent life in adulthood.

Despite the limitations they experience regarding in-person communication and social skills when relating to others, people with ASD have shown a penchant for technology [3]. Given the repetitive and prolonged nature of behavioral therapies for people with autism, technological interventions allow for visual and auditory environments (stimuli) this ensures the patience of people with autism with its recurrence. Moreover, the use of technology in behavioral therapy for people with ASD has the advantage of being cost-effective, both in terms of treatment center costs and caregiver costs [4]. Studies have also shown that people with ASD are better at working with visual stimuli compared with other sensory stimuli [5]. Based on the findings of these studies, technology as digital behavioral therapy has been applied in a number of ways. 

Augmented reality (AR) offers behavioral therapy in a way that enriches the experiences and skills of people with ASD, in addition to creating an integrated learning environment that provides for the possibility of three-dimensional (3D) visualization of learning content and the manipulation of physical objects [6]. Augmented reality enhances the imagination of ASD sufferers without negatively affecting it by creating ‘physical’ creatures to enhance selected targeted skills [7,8]. Moreover, using augmented reality, more interactive and attractive interfaces can be provided, removing the need for using traditional peripherals like the keyboard and the mouse [9]. 

One of the concepts integrated with digital behavior therapy technology is that of ‘serious games’. Serious games are used to measure the level of comprehension of an idea through a gaming environment to inspire motivation and commitment among people with ASD. Serious games provide many sensory stimuli, and studies have shown that it is possible to provide the attraction factor for participation through a framework or application that provides additional animation and images [10]. The technology provides a stimulating environment in which to effect traditional approaches, such as the Picture Exchange Communication System^®^ (PECS) methodology. The traditional PECS method employs physical cards, which work as a Basis of Verbal and social skills acquisition. Typically, parents begin to practice PECS with their children at the age of 5 years; however, PECS-based technology allows for this practice to start at a younger age. In this regard, graphic interfaces provide a better solution compared with the traditional approach, as it is simple and easy to navigate.

In this survey, we review the above areas in detail and, as such, this paper contributes to addressing the gaps and restrictions that exist in each of these fields. The essence of this review is to provide information about the latest studies conducted from 2015 to 2020 in the following categories: (1) studies that use an AR environment for assisting people with ASD in all skills; particularly, we focus on communication and social skills and take advantage of solutions attached to other skills; (2) studies that employ serious games and present specific standards that are used to develop skills (3); studies that include PECS applications and knowledge of how to apply the methodology with it six stages.

The rest of the paper is organized as follows. Section 1 reviews existing surveys related to field of research and the contributions in this survey. Section 2 provides background for each of the fields included in an AR environment, as well as serious games and PECS applications. Section 3 details the research method used in this Sys-tematic Literature Review (SLR). Section 4 presents the results of the reviewed studies. Section 5 presents a discussion on how to use the reviewed studies via the primary and sub-research questions. Section 6 summarizes ongoing issues and future directions. Finally, the paper is concluded in Section 7.

### 1.1. Related Surveys 

Caring for children with ASD has recently attracted broad interest in various fields, leading to researchers conducting numerous surveys on this topic. We identified selected areas in this context that were relevant to our survey as follows. Surveys related to AR combined with ASD; surveys that show the application of serious games with ASD; surveys that show applicatons used in PECS with ASD; recent surveys that bring all of these domains together with ASD. These surveys included the presentation of strategies and tools used in the specific fields in addition to additional research that focused on presenting the applications and systems used in treating ASD. Selected related surveys focused on the skills to be developed among children with ASD, followed by a comparison between these surveys with ours to identify the primary contributions of our work on this topic [11]. Revealed that most of the current AR applications that have been developed used four types of tools or strategies. Augmented reality applications rely on video modelling for visual cues, specific training and feedback, as well as final reinforcement components. The authors of [12] reviewed works targeting social interaction, emotion recognition, attention skills and functional learning using AR technology. The study found that AR techniques had a positive and effective impact on improving a range of areas, most notably social interaction, social communication skills, verbal/nonverbal communication, procedures for recognizing facial expression, attention skills and the career interests of children and adolescents with autism. The contributions of this survey appear to indicate the effectiveness of incorporating elements of AR in interventions aimed at improving the different skills of children and adolescents with ASD. 

The survey conducted in [13] presents a review of recent studies related to the use of AR for children and adolescents with autism as a means for learning a variety of skills. In addition, a research classification was proposed to evaluate AR technology among children and adolescents with autism. This assessment focused on nine aspects, i.e., learning skills, characteristics, symptoms, technology, research design/study type, data collection methods, settings, evaluation parameters and evaluation. The presented survey discussed in [14] is presented in two parts. The first addressed the basic characteristics and design criteria required for AR systems that aid in the diagnosis and treatment of children with autism. The challenges associated with this type of AR system were also reviewed. In the second part, the evaluations in the first part were used to develop a new AR system, as the system included most of the standards required to design an effective AR system for children with ASD. This system aims to diagnose early autism in children by measuring their upper limb movements, and it consists of two main components, an augmented reality game and a program to record movements. Using augmented reality, children are stimulated to simulate a virtual object to move their hands, and a Microsoft Kinect sensor records all the children’s movements for analysis. The analysis process relies on the support vector machine (SVM) and the extreme learning machine (ELM), through which the diagnosis of autism is reached. In [15] authors provide a survey of evidence-based AR empirical solutions developed for ASD that focused on improving social communication skills in 14 related studies. During which the authors concluded that the effectiveness of augmented reality in teaching social communication skills to an individual with autism is not possible, due to the heterogeneity of the participants and the great diversity of skills targeted in the relevant studies despite the positive effect in the use of AR in education for people with autism. In [16], an overview of the use of AR technology is presented from a patient’s perspective. By identifying the strengths and weaknesses of the methods used in 16 current studies. This study focused on reviewing many diverse technologies such as smart glasses, mobile devices and video display systems, and focused on abilities such as social skills, the ability to communicate, attention and cognitive skills. In [17], about augmented reality in autism treatment there is two aspects were identified that were found to be common to almost all current studies on ASD: (1) the high communication and cognitive performance of people with ASD; (2) experimental designs aimed at improving social skills, language and relationships. Adnan et al. [18] focused on the satisfaction and confidence aspect of using AR applications among children with ASD. 

The study found that using a visual approach in AR applications for training social interaction skills increased the enthusiasm and passion of ASD children. In [19], the purposes, limitations and effectiveness of AR and the quality and results of evaluation methods were analyzed among 10 recently published studies. These studies were divided into many categories based on content, social skills, emotion recognition and other factors. A central issue in the survey presented in [20] was determining which activities would be possible and most effective for applying the use of AR to develop techniques for ASD children and how to go about doing so. The authors note that few of studies recently published using different strategies (such as 3D cards) were directly related to approving treatments for ASD children.

On the other hand, for using the concept of serious games with ASD, three surveys were included in this area. In [21], the following meta-analyses revealed that limitations in applying the principles of serious game design may be computer-based socio-emotional interventions. Furthermore, a serious game assessment tool was developed to implement serious game principles when designing game-based interventions targeting ASD individuals. Studies such as [22] on the effectiveness of serious games for people with ASD deal with social skills, rather than targeting other adaptive behavior skills, since the field of serious game design has been successful in addressing many adaptive behavior skills and intellectual performance. Finally, through the survey presented in [23] measuring the effectiveness of information and communication technology and the use of serious games to treat people with ASD, authors Of the current study concluded that serious games incorporating ASD that are currently available have some limitations in terms of evidence of their clinical benefit.

In relation to PECS’s applications with ASD, as well as surveys that integrate the fields of AR, serious games and PECS’s technology with ASD, we found no surveys during the research process. Addressing these contexts represents one of the main contributions of the current survey. To identify all contributions, Table 1 provides a summary of previously published work and lists their limitations to identify the main contributions of the current survey.

### 1.2. Main Contribution of the Presented Work

Based on the previous review of the existing surveys, the current research presents the following primary contributions.

(1) Conducted a systematic and accurate survey of studies applying the PECS approach. Our goal is to understand the way of using PECS with ASD children.
(2) In contrast to existing surveys that focused on the number of targeted skills, as all previous surveys have demonstrated in related studies, and which primarily addressed social and communication skills, the current survey focused on improving and developing existing solutions. So we will try to review existing solutions using AR, SG and PECS with ASD children.
(3) Aimed to find common factors in the field of AR environments, serious games and PECS’s applications, and to illustrate how combining these fields can contribute to the existing body of research.
(4) Aimed to establish technical solutions for addressing different personality traits by defining specific criteria for people with ASD, as existing surveys did not consider solutions in terms of these differences and instead combined individuals with ASD with other groups (e.g., those with intellectual disabilities).

**Table 1:** Related surveys to the augmented reality environment and serious games with people with ASD.

Augmented Reality with ASD								
Title	Author	Journals	Year	Citation	Timeline	Literature Type	Num Selected Studies	Limitation
Technology-Assisted Intervention for Children with Autism Spectrum Disorder using Augmented Reality [11]	Suparjoh, Suriawati Shahbodin, Faaizah Ku, Che Che, Nuraini Mohd, Ku	International Journal of Recent Technology and Engineering	2020	0	2012 to 2018	systematic literature review	13	This SLR represents a presentation of previous studies without the opinions or criticism of researchers for these studies
Exploring the Impact of Augmented Reality in Children and Adolescents with Autism Spectrum Disorder: A Systematic Review [12]	Berenguer, Carmen Baixauli, Inmaculada Gómez, Soledad Andrés, María de El Puig De Stasio, Simona	International Journal of Environmental Research and Public Health	2020	0	2010 to 2020	systematic literature review	20	The low number of high-quality designs that have been implemented in studies as they are limited to communications and/or posters at international conferences and experimental studies. The characteristics of the sample, as it contained samples from children and adolescents with high-performance autism and did not take into account the greater heterogeneity of the disorder in order to generalize the results. A lack of longitudinal investigations to find out whether these techniques might really help children with autism improve social interactions. of augmented reality are considered.
Augmented reality for learning of children and adolescents with autism spectrum disorder (ASD): A systematic review [13]	Khowaja, Kamran Banire, Bilikis Al-Thani, Dena Sqalli, Mohammed Tahri Aqle, Aboubakr Shah, Asadullah Salim, Siti Salwah	IEEE Access	2020	4	2005 to 2018	systematic literature review	30	------------------------------
The use of augmented reality in the diagnosis and treatment of autistic children: a review and a new system [14]	Wedyan, Mohammad AL-Jumaily, Adel Dorgham, Osama	Multimedia Tools and Applications	2020	1	n.d.	literature review	24	-------------------------------
Use of augmented reality for social communication skills in children and adolescents with autism spectrum disorder (ASD): A systematic review [15]	Khowaja, Kamran Al-Thani, Dena Banire, Bilikis Salim, Siti Salwah Shah, Asadullah	ICETAS 2019–2019 6th IEEE International Conference on Engineering, Technologies and Applied Sciences	2019	1	2005 to 2018	systematic literature review	14	1-The need for a longitudinal study. Also, researchers need to determine the number and duration of sessions.
Using Augmented Reality in Patients with Autism: A Systematic Review [16]	Marto, Anabela Almeida, Henrique A. Gonçalves, Alexandrino	Lecture Notes in Computational Vision and Biomechanics	2019	5	n.d.	systematic literature review	16	1-The effect of individual differences in the study.2-Small sample sizes from 1 participant, up to 12.
New Technologies and Autism: Can Augmented Reality (Ar) Increase the Motivation in Children With Autism [17]	Rega, Angelo Mennitto, Andrea Vita, Salvatore Iovino, Luigi	INTED2018 Proceedings	2018	9	n.d.	systematic literature review	14	------------------------------
Systematic review on augmented reality application for autism children [18]	Adnan, Nur Hidayah Ahmad, Ibrahim Abdullasim, Nazreen	Journal of Advanced Research in Dynamical and Control Systems	2018	0	2012 to 2018	systematic literature review	5	1-The effect of generalising potential for improvement remains to be examined using systematic intervention methods over a longer period.2-Engaging autism to explore the mechanism of augmented reality.
The Application of Augmented Reality for Intervention to People with Autism Spectrum Disorders [19]	Karamanoli, MSc Persefoni	IOSR Journal of Mobile Computing & Application	2017	1	2012 to 2016	Literature Review	10	The methodology followed is imprecise and not enough clear
Augmented Reality and the Use of Alternative Communication for Children with Autism Spectrum Disorder: A Literature Review [24]	Marcelo Marcio Soares(&) and Aline da Silva Oliveira Neves	springer	2020	0	2013 and 2018	systematic literature review	5	few studies are studied and analysed
Serious Games with ASD								
Title	Author	Journals	Year	citation	Timeline	Literature type	Num selected studies	Limitation
A systematic review and meta-analysis of social emotional computer based interventions for autistic individuals using the serious game framework [21]	Tang, Julia S.Y. Chen, Nigel T.M. Falkmer, Marita Bölte, Sven Girdler, Sonya	Research in Autism Spectrum Disorders	2019	4	1990 to 2018	systematic review and meta-analysis	17	----------------------------
Studying the effects of computer serious games on people with intellectual disabilities or autism spectrum disorder: A systematic literature review [22]	Tsikinas, Stavros Xinogalos, Stelios	Journal of Computer Assisted Learning	2019	22	2005 to 2018	systematic literature review	58	-------------------------------
Serious games to teach social interactions and emotions to individuals with autism spectrum disorders (ASD) [23]	Grossard, Charline Grynspan, Ouriel Serret, Sylvie Jouen, Anne Lise Bailly, Kevin Cohen, David	Computers and Education	2017	64	2001 to 2014	systematic literature review	31	-------------------------------

## 2. Background

### 2.1. Autism Spectrum Disorder (ASD)

**Figure 1:**
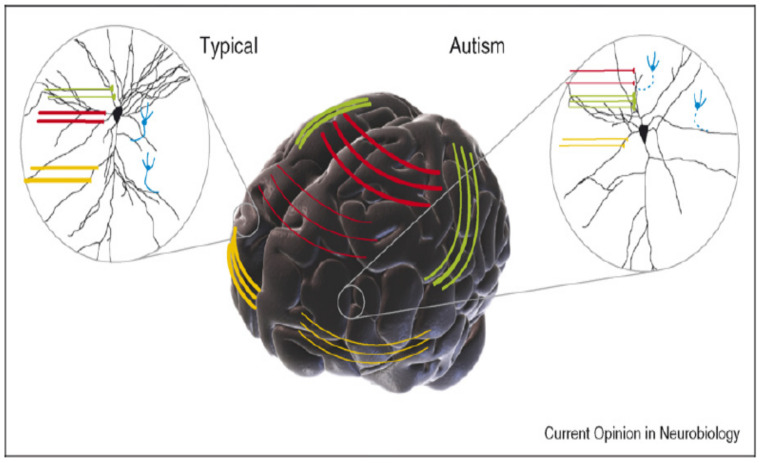
Definition of ASD.

There are many definitions of ASD, all of which canter on the notion that ASD can be described in the simplest terms as impairment in social communication that develops early during childhood and is caused by a heterogeneous set of neurodevelopmental conditions, such as repetitive sensory and motor behaviours that are associated with a strong genetic component [25,26,27,28]. Autism can be defined in terms of the pathological anatomy of the cerebral cortex. It involves only a slight disturbance in the basic and diamond radial organization of neurons and glia in this particular area of the brain [29]. Studies have expressed it as a slight disorder that is extremely variable in dendritic orientation and in the decrease in size and spacing between the small radial columns of neurons in different cortical regions, including the frontal lobe [30,31]. As shown in Figure 1, small for some mandibular tracts (indicated by the red lines) and anterior temporal joints (indicated by the yellow lines) and are responsible for stabilizing human contact. However, the severity and size of the disorder may differ between people; accordingly, different types of autism present with different characteristics as shown in Table 2 [18].

This survey focuses on all autism types except Asperger’s syndrome and pervasive developmental disorder not otherwise because these represent severe disorders that require complex clinical procedures. 

Although there is no drug treatment for autism spectrum disorder, early treatment can make a significant difference in the lives of children with autism [32]. Following the diagnostic process by a specialist, a treatment method will depend on determining the severity of the disorder and the child’s age. Typically, the earlier treatment can be started, the better the results will be. There are many therapeutic approaches that ASD children can practice at home, in school and in specialized clinics. Specific types of treatment methods can be presented as follows: behavior and communication treatments, applied behavior analysis, educational therapies, family therapies, speech therapy, occupational therapy, social skills classes, hippo therapy and using the PECS [33].

**Table 2:** Describe the types of autism [18].

Type of Autism	Describe
Autistic disorder	Abnormal or impaired development of social interaction and a limited repertoire of activities and interests, usually noticed during the first years of life
Asperger’s syndrome	Severe and persistent impairment of social interaction and development of patterns of behavior, interests, and restricted and repetitive activities. No clinically significant delays in language acquisition
Rett’s disorder	A very specific and distinct pattern of stunted growth after a period of normal functioning during the first five months after birth. Diagnosed only in females
Childhood disintegrative	Decline in multiple areas of function after a period of at least two years of apparent normal development
Pervasive developmental disorder not otherwise	Severe and pervasive impairment in the development of social reciprocal interaction, but criteria for a specific pervasive developmental disorder are not met.

### 2.2. The Picture Exchange Communication System

**Figure 2:**
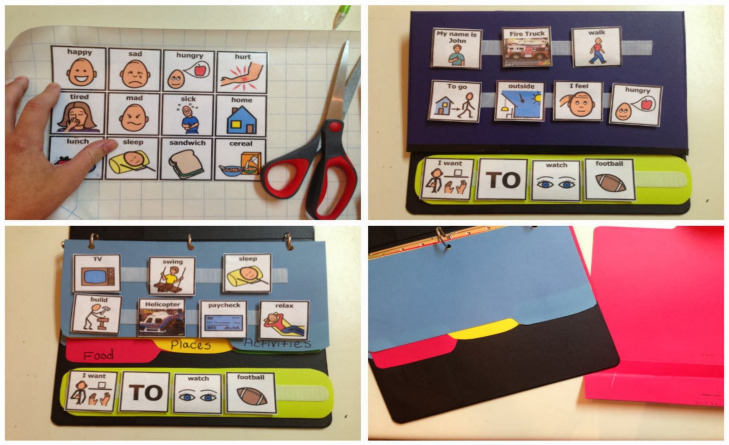
Materials for creating a PECS system.

As previously noted, one of the methods of treating ASD is the PECS. The PECS system was developed by Andy Bundy and Laurie Frost in 1994 [34,35]. It is a communication system that mainly depends on exchanging images to express basic needs and communicate with others [36,37]. The PECS system targets children with a nonverbal autism spectrum disorder. It works using the exchange of well-known signals, images and symbols to enable effective communication as one of the Augmentative and Alternative Communication (AAC) strategies [38]. The PECS approach has the advantage of being a system supported with outdoor equipment at low technical costs because it relies only on painted drawings and recording the images in these drawings [39] as shown in Figure 2. 

Furthermore, PECS provides more tangible reinforcement, as children receive tangible objects in its first stage, which teaches how to order first, which in turn enhances dealing with the components of the surrounding environment. Furthermore, PECS is distinguished by the fact that it does not require a child to have existing communication skills to learn about communication. Research by Bundy and Frost demonstrated that 59% of ASD children who were trained using PECS spontaneously developed independent speech [40].

The PECS approach comprises six stages. In the first stage, a picture is exchanged to discover how to deal and obtain the required item through pictures and this process develops until it reaches the sixth stage, in which simple questions and mutual comments are answered [41]. Table 3 briefly explains each of these stages. The PECS approach functions as per guidelines established by [34].

**Table 3:** Stages of PECS.

Stage Name	Description	Outcome
How to Communicate	Children learn to exchange pictures of objects or activities they desire.	Training to take the picture from the table and put the image in the hands of the communication partner.
2. Distance and Persistence	Children learn to generalize this new skill if they continue to use individual images in different places and with different individuals. They are also taught to be more persistent and persistent in communication.	He goes to the profile, grabs the photo, goes to the communication partner, gets attention, and leaves the picture in hand.
3. Picture Discrimination	Children learn to choose between two or more pictures to order the things they want.	To ask for the things he wants by moving to the communication book and choosing the appropriate image from among several images
4. Sentence Structure	Children learn to attach a simple sentence to tape using the picture “I want” followed by a picture of the required thing.	To request existing and non-existent things using multi-word phrases by navigating to the communication file and taking a picture (I want) + (picture of the desired thing) and placing it on the sentence bar and taking the sentence bar from the communication file and directing it to the communication partner and giving it to him
5. Answering Questions	Children learn to use PECS to answer the question, “What do you want?”	To automatically ask for many things and to answer the question (What do you want?)
6. Commenting	Children are now learning how to comment in response to questions such as: “What do you see?”, “What do you hear?” And “What is this?” They also learn how to construct sentences starting with “I see”, “hear”, “feel”, “it”, and so on.	To answer (What do you want?) (What do you see?) (What do you hear?) (What is this?) And automatically ask and comment on events he sees.

### 2.3. Serious Games

**Figure 3:**
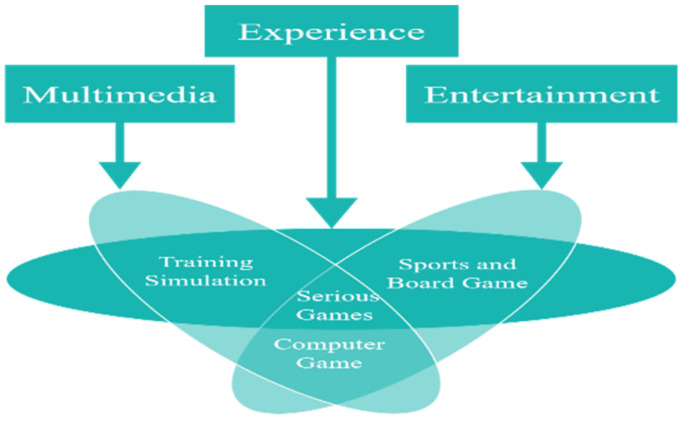
Definition of serious games (as presented in [40]).

Other concepts that can be incorporated into ASD treatment is that of serious games. Although several different definitions can be applied, “serious games” involve many different types including training simulations, computer games and sports; serious games can be defined as digital games with several purposes, such as providing an experience of multimedia, as opposed to simply serving as entertainment [42]. They can also be defined as games that are designed with a primary purpose other than entertainment. Moreover, some [43] posit that the term “serious” in serious games refers to messages or input that is part of in-game content aimed at providing knowledge or training skills, i.e., providing the player with knowledge or experience management in a suitable environment [43]. To identify that serious games involve applications comprising experience, entertainment and multimedia, through the previous definition, it is clear that serious games are the result of the merging of three concepts, which are training simulation, computer games, and sports. As shown in Figure 3. Serious games can be combined with the latest technologies to contribute to increasing their benefits and purpose.

The rapid development of serious games is expected to increase in the future [44,45]. To ensure the success of serious games and to apply them to their full extent, several criteria that contribute to their benefits must be considered [43]. Five of these criteria include activity, modality, interaction style, and environment and application area. ‘Activity’ refers to determining the type of activity for which a game was designed. Many types of activity, including physical activity, target health and movement activities such as recreational games [46,47] and games aimed at obese children [48]. Another type of activity is physiological, which can be used in rehabilitation games [49] and to diagnose health conditions [50]. The activity can also be mental, i.e., one that is used to learn [51,52] and provide training in different fields. The ‘modality’ criterion refers to the senses that are used to transfer information from a device to the game participants. The most common senses in this regard are sight, hearing and touch, with recent attempts to include the olfactory sense [53]. To use serious games to their full extent, attempts have been made to integrate a wide range of senses in their mechanics, e.g., enabling participants to mix music or engage in tactile experiences [54]. The ‘interaction’ criterion refers to the specific input used within the game environment to enable participant interaction; these include traditional inputs, such as a keyboard, mouse and joystick, as well as more modern and smart inputs, such as enabling tactile experiences (touch) and tracking eye gaze and movement using sensors. Correctly selecting entries together with game requirements increases the success and effectiveness of serious games. One example that provides a good description of the input used for interaction was explored in [55] (Whitehead et al. 2010), i.e., the Sensor Network for Active Play system. This example involved a game aimed at effecting physical activity by connecting various sensors to the feet and arms of a game user. 

**Figure 4:**
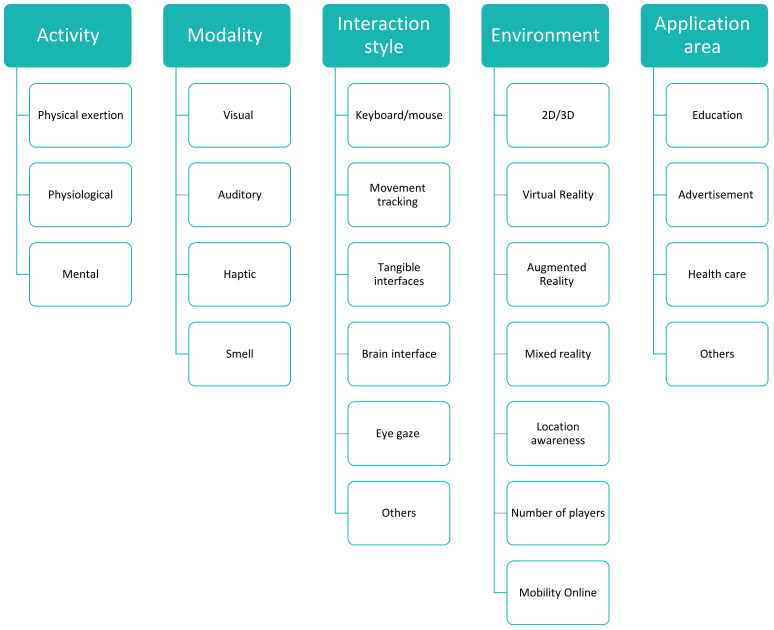
Serious Games Standards.

The ‘environment’ criterion refers to the type of digital environment used in a game. Different types of serious game environments can be combined. The environment may be a two-dimensional (2D) or 3D game, or a combination of both. Alternatively, the game environment can employ an augmented, virtual or mixed reality. These types will be defined in more detail in the next section. The AR or virtual reality is widely used in serious games such as those defined in [54]. Mixed reality is also discussed in [48]. This criterion addresses whether the game requires permissions to locate the player. It also determines whether the game is portable. In addition, the criterion also determines whether play is affected online, in addition to the number of players in the game and whether it is individual or collective as discussed in [47]. The fifth and final criterion that must be determined in the design of serious games is the ‘application area’, which refers to serious games’ target areas for application. These include education and advertising, which dominate serious games by up to 57% according to [56], while the remaining percentage relates to less frequently addressed areas such as healthcare, rehabilitation, communication between people, luxury, tourism, antiquities and others. Figure 4 briefly illustrates the primary criteria for serious games.

### 2.4. Augmented Reality

**Figure 5:**
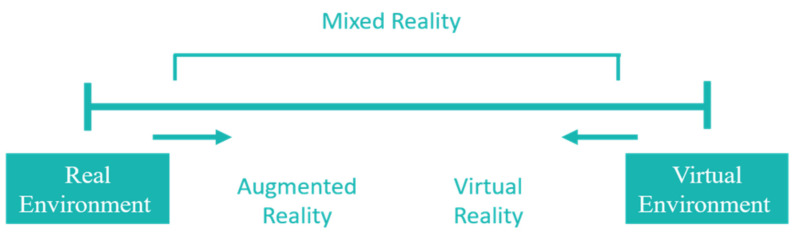
Reality-virtuality continuum (as presented by [59]).

One of the most important environments that can be used in serious games is known as AR. In the 1960s, AR prototypes were invented by Evan Sutherland and his students, in which code was used to display 3D graphics. Augmented reality was first recognized as an emerging technology in 2007 [57]. The technology involves 3D interactive recorded data that combines real and virtual objects [58]. Figure 5 (as presented by [59]) illustrates that AR is part of a connected chain that extends between real and virtual environments and includes AR and augmented virtual reality (AVR), where AR is closer to the real world and AVR is closer to the purely virtual environment. Two main types of AR can be defined as follows:

- Location-based AR, which depends on devices or mobile phones that have a Global Positioning System (GPS) to display digital media as discussed in [60];
- Visual-based AR, which depends on the user’s phone camera orientation to the object in the digital environment [61,62].

These two types of AR must, however, be able to preserve the shape of virtually created elements that merge with elements in reality, thereby rendering them compatible with the real environment. Furthermore, the performance of the virtual environment should be effective in real-time [63,64].

Augmented and AVR are often confused because while they are almost the same, there are subtle differences between them. In an AR, the user reference diagram is linked to reality but additional technical elements are added, making it appear as if these items are default aspects of reality. In an AVR environment, the user reference diagram is linked to a completely virtual world, and the player uses a virtual environment to engage in a particular experience [63,65,66]. Table 4 shows the most important differences between AR and AVR [14,67,68].

**Figure 6:**
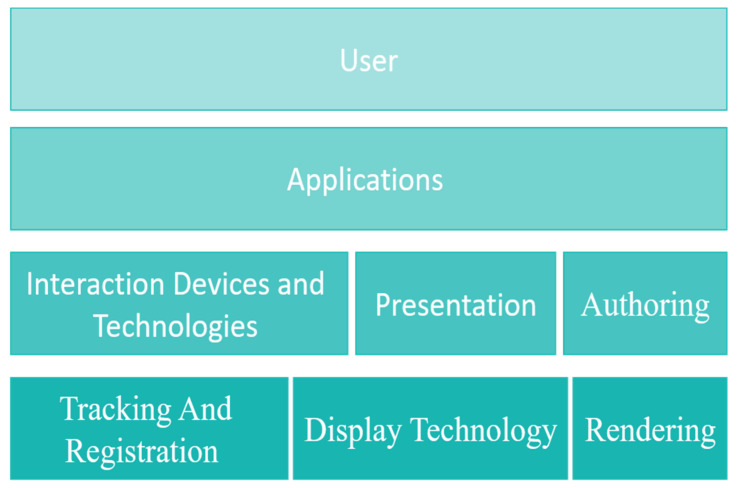
The component of AR.

Augmented reality operates within a framework that includes three main phases. In the first stage, tracking, recording, display technologies and processing processes take place in real-time. This stage is important for creating the virtual object. The second stage includes interaction devices and procedures, in addition to presentation and configuration processes. The third stage is the application stage and includes the user interface. Figure 6 illustrates the different components of AR [69,70].

## 3. Review Methodology

**Figure 7:**
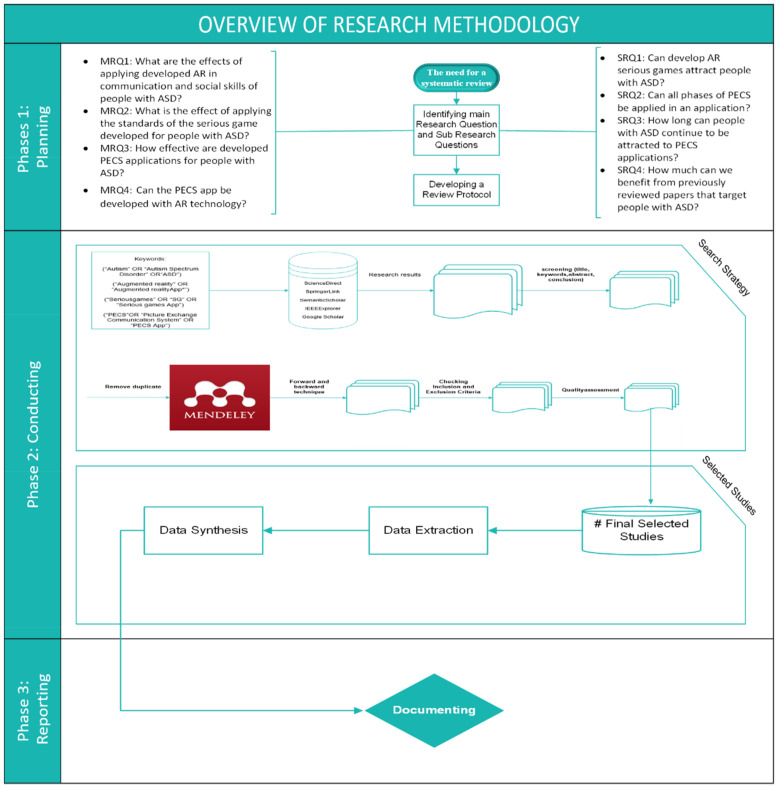
Overview of the research methodology.

This survey follows the methodology of a systematic review to examine current research work. A comprehensive view of recent proposals for rehabilitating children with ASD is provided and serves as a means for examining and evaluating current studies relevant to the topic to provide comprehensive information to the research community in several areas. In addition, this survey delivers a new proposition by developing the limitations of the current studies. This survey was conducted by following the recommended guidelines accordingly [71]. The survey process comprised three phases, i.e., planning, conducting and reporting. Figure 7 summarizes the steps required for a systematic review [72]. Details of these steps are explained in the following sections.

### 3.1. Planning the Review

#### 3.1.1. The Need for a Systematic Review 

**Figure 8:**
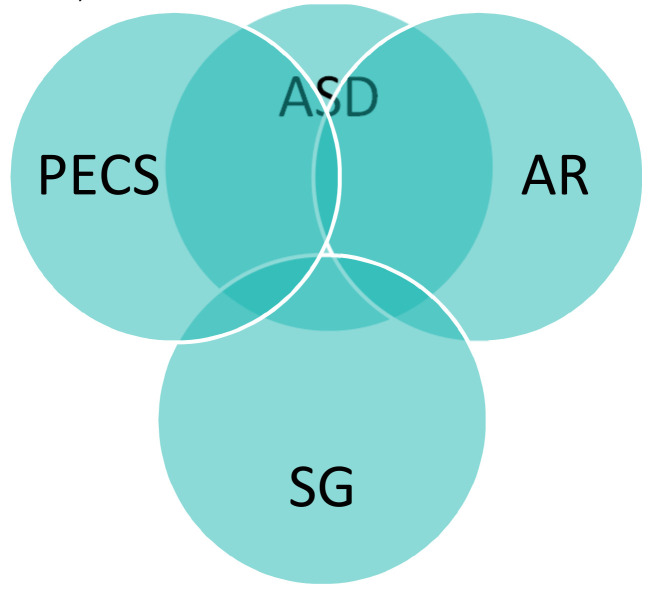
Overlapping domains of ASD patient rehabilitation, augmented reality environment, serious games, and PECS applications.

Although many applications have been proposed in the field of rehabilitation for children with ASD, many technical interventions that contribute to increasing rehabilitation effectiveness are still required. This study reviewed several ways in which to develop rehabilitation for children with ASD by integrating approaches into three domains, i.e., augmented reality, serious games and the PECS approach. These areas were previously individually suggested in the reviewed literature; the current survey broadens the discussion and investigates integrating the suggested areas according to the following research questions [72]. Figure 8 shows the current relationship between these fields, considering the lack of research that combines them.

#### 3.1.2. Defining the Research Questions

To achieve the main objectives of this study, the PICOC method [72] was used to define the primary and sub-research questions. The main research questions are as follows.

(1) MRQ1: What are the effects of applying developed AR in the communication and social skills of people with ASD?
(2) MRQ2: What is the effect of applying the standards of serious games developed for people with ASD?
(3) MRQ3: How effective are the developed PECS applications for people with ASD?
(4) MRQ4: Can the application of the PECS be developed using AR technology? Additionally, the following sub-research questions are proposed.
  - SRQ1: Can developing AR serious games attract people with ASD?
  - SRQ2: Can all the phases of PECS be implemented in a mobile application?
  - SRQ3: How long can people with ASD continue being attracted to the application of PECS?
  - SRQ4: How much can we benefit from previously reviewed papers that target people with ASD?

#### 3.1.3. Review Protocol Development

The protocol development process contributes to the process of identifying the methods used in the systematic review components (review background, MRQ and SRQ development, search strategy, inclusion and exclusion criteria, data extraction and data synthesis). 

The result of the review protocol development process aimed to reduce study bias and distinguish between SLR and traditional methods of reviewing the literature [73,74,75].

### 3.2. Conducting the Review

**Figure 9:**
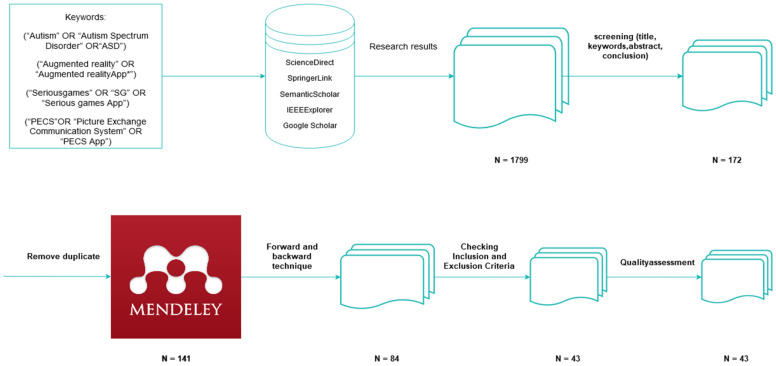
Search Strategies.

Search strategy: As shown in Figure 9, the search strategy aimed to extract data from the relevant research papers. Accordingly, a search strategy must be developed to ensure that scholars obtain the largest possible number of relevant studies. To ensure that additional academic publications are included, manual and automatic research methods were used, in addition to examining the content included in the review. An automatic search was used to find preliminary studies related to people undergoing ASD rehabilitation. The automatic search of library databases via the Internet was conducted based on search keywords. The following online databases (with their accompanying website link) were included in the search strategy:

- Science Direct (www.sciencedirect.com, accessed on 13 December 2021)
- Springer Link (www.linkspringer.com, accessed on 13 December 2021)
- Semantic Scholar (www.semanticscholar.org, accessed on 13 December 2021)
- IEEEXplorer (www.ieee.org, accessed on 13 December 2021)
- Google Scholar (www.scholar.google.com, accessed on 13 December 2021)

The proposed steps in [13] were followed to derive the search terms as follows.

Seeking keywords using the main and sub-research questions, four main keywords/phrases were identified (autism spectrum disorder, augmented Reality, PECS, serious games).

(1) Alternate spellings, synonyms and abbreviations for each keyword were defined.
(2) The keywords used in the relevant searches were defined.
(3) Similar keywords were defined and divided into four categories for each of the relevant proposed domains so that the common category for all domains was ‘autism spectrum disorder’.
(4) Used the OR logical operator to combine keywords in each category.
(5) Using the AND logic operator by combining keywords across categories.
(6) Each of the search rules differed in terms of filtering searches; Table 5 shows the specifically applied filtering and the particular query formed from the keywords.

**Figure 10:**
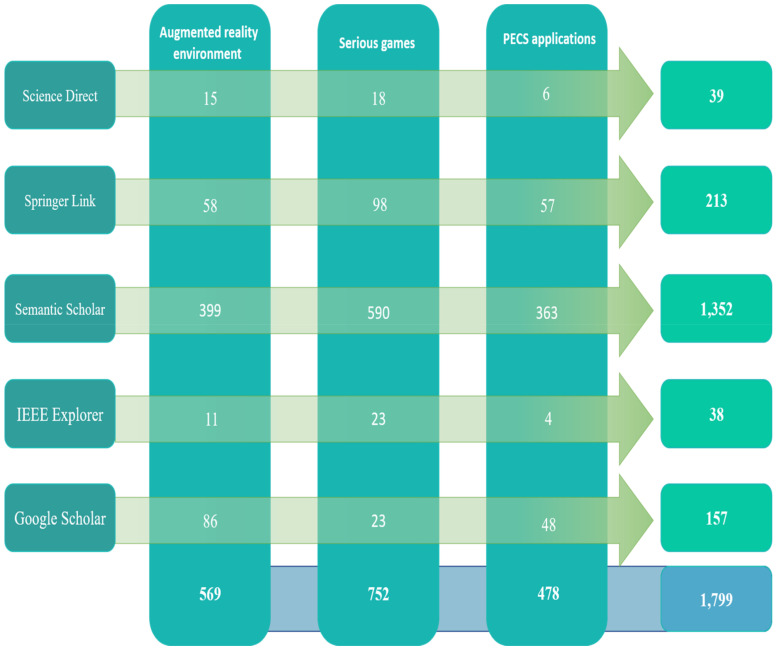
Search results from search databases.

The search was restricted to the period 2015 to 2020 and found a good amount of literature within the three different fields. The total number of studies related to an AR environment was 569; 752 studies were found in the field of serious games and 478 were derived in the field of PECS applications. All of the documents identified were research articles. Next, all library databases were manually searched using the pre-defined keywords. Details of the overall search are shown based on the keywords identified in the libraries (identified in Figure 10).

### 3.3. Criteria for Including and Excluding Articles

From a search of five databases, 1799 related studies were found. Next, incorrect quotes screening reduced studies related by title, keywords, abstract and conclusion to 172 studies. Next, 31 duplicates were removed to reduce the related studies to 141. Using the forward and backward technique, the number of studies was further reduced to 84. First, the title of each study was considered. Next, the content of each primary study was reviewed starting with the abstract. The primary studies incapable of addressing one or more of the RQs related to this SLR were excluded from the list of related studies. Next, an analysis of the remaining studies was carried out based on the selection criteria. Then, inclusion and exclusion criteria (see Table 6) were applied as part of the selection criteria to reduce the number of included studies. The collective selection process reduced the related studies to 43. The references section of each remaining study was then manually checked to identify additional related studies but did not yield additional new and related studies. Finally, a qualitative assessment of the primary studies was performed; no additional new and related studies were found. The qualitative assessment of related studies is described below.

**Table 6:** Criteria for inclusion and exclusion of the articles.

Inclusion Criteria	Exclusion Criteria
Studies are written in English only	Studies for which the full text is not available
Studies have been published from 2015 to 2020	Duplicate studies
Studies type is Journal articles and conference papers.	Studies that do not directly aim to help people with autism spectrum disorder only (without other disabilities).
All methodology except (Review, Survey)	Studies involving VR and MR in studies related to augmented reality with ASD.
Studies that provide answers to the main research question or the sub-research questions	Studies that include all types of game-based learning and do not specialize in serious game.
	Studies not related to the applications of PECS

*Manual Search*

The citations to related studies were traced using the forward and backward technique in [76,77], and the included databases were searched to find the studies cited in the selected studies. Among others, the Mendeley tool was used to sort and manage all studies and to remove duplicates. Using the manual search step, a systematic review of the research was confirmed as complete and relatively comprehensive.

*Selection of Studies*

**Figure 11:**
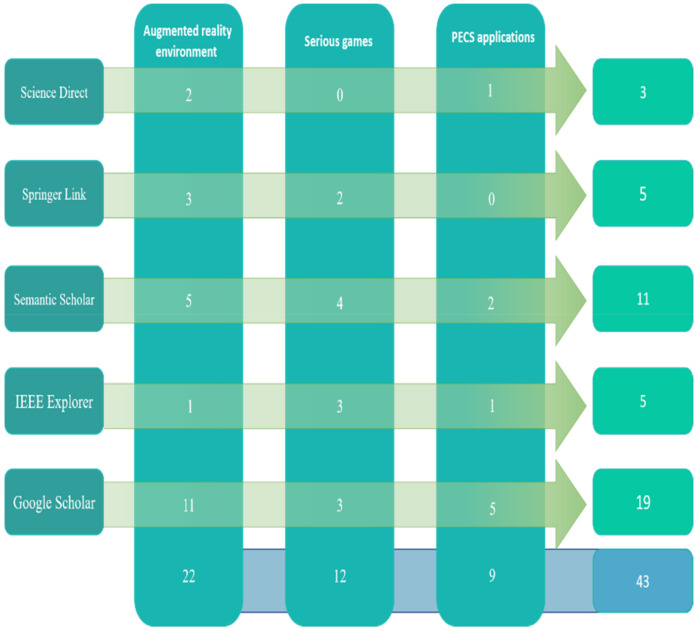
Filtering with Checking Inclusion and Exclusion Criteria.

As shown, using the steps for effecting automatic searches, 141 related studies were found. In the manual search, all articles were checked using the Mendeley tool. Duplicate articles were removed by checking the titles of each article. As a result, a total of 121 articles were obtained. The focus then moved to reviewing the summary of each article, which subsequently included 98 articles. In the final step, these articles were examined by focusing on their content, reducing the overall number to 43. Figure 11 shows the stages for selecting articles, based on checking the inclusion and exclusion criteria.

*Quality Assessment of Primary Studies*

Quality assessment (QA) was performed for each study to measure the quality of the content presented. A set of quality assessment questions was created to determine the suitability of the related studies.

(1) QA1: Are the research goals/objectives clearly defined?
(2) QA2: Is the solution based on AR/serious games/PECS clearly defined?
(3) QA3: Is the research methodology specified in the article?
(4) QA4: Are the search results reported?

The above four QA criteria were tested for the 43 included research papers to determine their reliability. The QA comprised three quality schema stages, i.e., high, medium and low, in which the quality of each paper relied on its loading score. For example, papers that satisfied the criteria were awarded a score of 2, papers that partially satisfied the criteria were awarded a score of 1 and papers that did not provide any information regarding the question and did not satisfy the criteria were awarded a score of 0. Consequently, based on the four QA defined criteria, studies with a score of 6 or above were considered to be of high quality, studies with a score of 5 or above were considered to be of medium quality, and studies with a score below 4 were considered to be of low quality. Table 7 presents the QA list for each study.

**Table 7:** Qualitative assessment of the related studies.

Augmented Reality						
Paper #ID	Article_ID	QA1	QA2	QA3	QA4	Score
[78]	A_1	2	2	2	2	8
[79]	A_2	2	2	2	2	8
[80]	A_3	2	2	2	2	8
[81]	A_4	2	2	1	0	5
[82]	A_5	2	2	2	2	8
[83]	A_6	1	1	1	1	4
[84]	A_7	2	2	2	2	8
[85]	A_8	2	2	2	2	8
[86]	A_9	2	2	2	2	8
[87]	A_10	2	2	0	1	5
[88]	A_11	2	2	0	2	6
[89]	A_12	2	1	1	1	5
[90]	A_13	2	2	2	1	7
[91]	A_14	2	1	2	2	7
[92]	A_15	2	1	1	2	6
[93]	A_16	2	1	1	2	6
[94]	A_17	2	2	2	2	8
[95]	A_18	2	2	1	2	7
[96]	A_19	2	2	1	0	5
[97]	A_20	2	1	2	2	7
[98]	A_21	2	1	2	2	7
[99]	A_22	2	2	2	2	8
Serious games						
Paper #ID	S_ID	QA1	QA2	QA3	QA4	Score
[100]	S_1	2	1	1	2	6
[101]	S_2	2	2	1	0	5
[102]	S_3	2	2	2	1	7
[103]	S_4	2	1	2	2	7
[104]	S_5	2	1	2	2	7
[105]	S_6	2	1	1	2	6
[106]	S_7	2	0	2	2	6
[107]	S_8	2	2	1	2	7
[108]	S_9	2	2	2	1	7
[109]	S_10	2	2	2	2	8
[110]	S_11	2	1	2	2	7
[111]	S_12	2	2	2	2	2
PECS						
Paper #ID	P_ID	QA1	QA2	QA3	QA4	Score
[112]	P_1	2	1	2	2	7
[113]	P_2	2	1	2	2	7
[114]	P_3	2	1	1	1	5
[115]	P_4	2	2	2	2	8
[116]	P_5	2	1	1	1	5
[117]	P_6	2	1	2	2	7
[118]	P_7	2	2	2	2	8
[119]	P_8	2	2	2	2	8
[120]	P_9	2	0	2	2	6

*Data Extraction and Synthesis*

A set of guidelines described in [121] and related to the data extraction process was followed to determine the relevant information from the related studies The data extraction method was designed to accurately record all information for each study in a summary. Features were determined considering all the research questions related to the systematic review.

## 4. Relevant Research

### 4.1. Combining an Augmented Reality Environment with Autism Spectrum Syndrome

A summary of related studies in the AR field, based on publication year, study type, country, participants, evaluation time, intervention, research strategy, methodology, device type, skills targeted and limitations are presented in Table 8.

**Figure 12:**
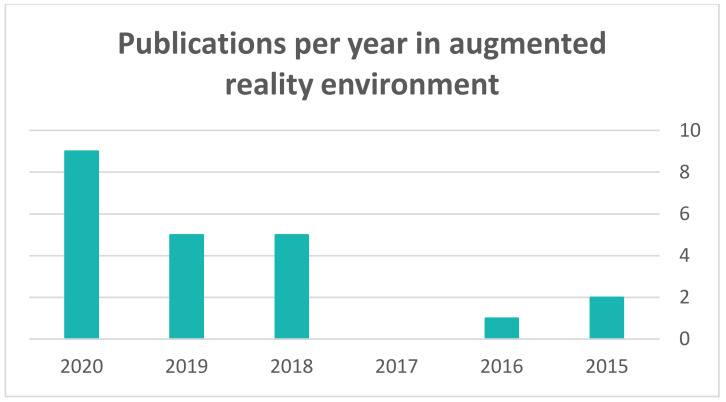
Publications per year in augmented reality environment.

Figure 12 shows the number of studies published between 2015 and 2020. As is evident, a number of studies were recently published due to the growing interest in AR technology. This interest was reflected in the announcements of large companies supporting AR technology in their products (e.g., Apple).

Among the related studies, 36% were published in journals, while the largest percentage was published as part of conference proceedings (64%). We note that the percentage of studies published as part of conferences was greater than the percentage of studies published in journals. We anticipated this to have been due to limitations that will be discussed in detail in the section on ongoing issues, which could be bypassed and published in conferences.

Through deeper analysis of the relevant studies, the following results emerged.

**Figure 13:**
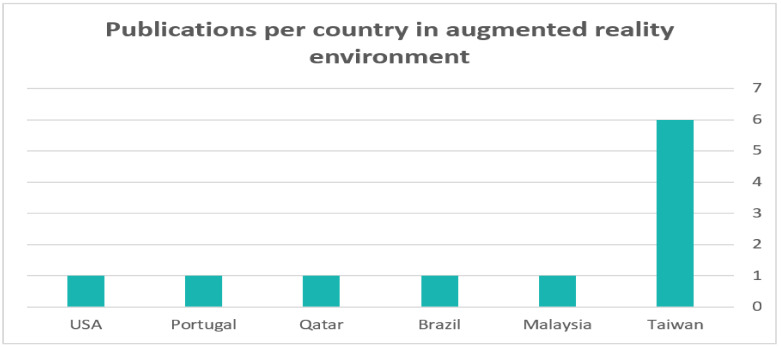
Publications per country in augmented reality environment.

- Figure 13 shows the number of included studies per country. The bulk of these works were from Taiwan (six published studies), followed by Malaysia, Brazil, Qatar, Portugal and the United States (one study per country); 11 studies did not clarify their country of publication. This indicates that researchers in Taiwan are interested in integrating AR in the rehabilitation of people with ASD and may also be attributed to the increased prevalence of autism. Based on a World Health Organization (https://www.who.int/news-room/fact-sheets/detail/autism-spectrum-disorders, accessed on 13 December 2021) report, the prevalence of autism worldwide is between 1 and 6. This prevalence is rated between 2.8% and 4.1% in Taiwan [122].

**Table 8:** Related work of using AR with ASD.

A_ID	Year	Country	Evaluation Time	Intervention	Research Strategy	Methodology	Device Type	Skills Targeted	Limitations
A_1 [78]	2016	Taiwan	4 weeks	Video-modeling storybook	Experiment	Qualitative	Tablets	Facial expressions	A bias in choosing participants in the experiment.
A_2 [79]	2018	n.d.	n.d.	n.d.	Experiment	Qualitative	Mobile devices	Social communication skills and cognitive skills	They received all 4 applications and their interactions with AR applications were during one day. There are no sound effects.
A_3 [80]	2020	n.d.	8 weeks	Social story and sequence learning approach	Experiment	Qualitative	Tablets	Social communication skills	To use the system requires physical community storybooks and script cards
A_4 [81]	2020	Taiwan	5 weeks	Visual framework of the concept map (cm)	Experiment	Qualitative	Computers	Social communication skills	There are several physical setup for the training system
A_5 [81]	2020	Qatar	n.d.	n.d.	Experiment	Qualitative	Mobile devices	cognitive skills	You need a caregiver’s intervention in determining the level of a game based on the individual’s need and level.
A_6 [83]	2020	n.d.	7 days	Kinect sensor	Experiment	Qualitative	Computers	n.d.	
A_7 [84]	2015	Portugal	n.d.	Alternative communication (pecs concept in some stages) and applied behaviour analysis strategies	Experiment	Qualitative	Computers	Social communication skills	The system needs the correct cards to work
A_8 [85]	2019	n.d.	n.d.	Optical see-through (ost)	Case study	Qualitative	Head-mounted devices	Facial expressions	Occluding real-world objects is not one of the strengths of ost ar displays. The design works better in indoor settings without strong light coming through the headset. Users with different pupil distances might experience differences in the depths of the rendering in 3d space.
A_9 [86]	2015	Taiwan	1 month and 2 weeks	A self-facial modeling learning system	Experiment	Qualitative	Computers	Facial expressions	It’s a complicated process while creating 3d characters. The number of participants is small
A_10 [87]	2019	Taiwan	n.d.	Key partial video with action Auto organizational menu (aom)	Experiment	Qualitative	Smartphones	Social communication skills	The number of participants is small. The results are unclear The complexity of scanning shapes to represent them with ar
A_11 [88]	2018	n.d.	n.d.	Books	Case study	Qualitative	n.d.	cognitive skills	The ambiguity of the research methodology It did not clarify what is new in it
A_12 [89]	2020	n.d.	n.d.	Deep convolutional neural networks	Experiment	Qualitative	Smartphones	Facial expressions	It is impractical in the communication process as users will need to place their phone cameras and point them towards whomever they are talking to. Participant details and duration of the experiment were not shown.
A_13 [90]	2019	n.d.	n.d.	Discrete trial teaching (dtt) approach	Case study	Qualitative	Smartglasses	Daily living skills	The system has not yet been evaluated to verify the extent to which autistic children acquire certain skills after using the system.
A_14 [91]	2020	Taiwan	18-week	n.d.	Experiment	Qualitative	Smartphone	Facial expression	They do not support independence and require a physical therapist for interaction and encouragement processes
A_15 [92]	2018	n.d.	n.d.	n.d.	Case study	Qualitative	Mobile devices	cognitive skills	There are many technical glitches that make the use of the application restricted with some restrictions
A_16 [93]	2018	Usa	3-week	Face2face module	Case study	Qualitative	Smartglasses	Social communication skills	The face2face unit is not a precisely described unit that contains a series of levels and difficulty settings
A_17 [94]	2018	Taiwan	Four months and two weeks	Concept map (cm) strategy	Experiment	Qualitative	Tablets	Social communication skills	Gestures and facial expressions are not designed for avatar
A_18 [95]	2018	n.d.	n.d.	StorybookLeap motion controller	Experiment	Qualitative	Computers	Social communication skills	Using computers that require many other devices to read the marker story
A_19 [96]	2020	Brazil	3 years	Pecs concept	Experiment	Qualitative	Mobile devices	cognitive skills	No numbers are showing the results of the trial during the intervention phase.
A_20 [97]	2019	Malaysia	n.d.	Phonetic reading system	Experiment	Qualitative	Mobile devices	cognitive skills	Difficulty scanning the ar marker card due to the vibration of the mobile device or the light
A_21 [98]	2020	n.d.	n.d.	Kinect skeletal tracking (kst) system	Experiment	Qualitative	Computers	Social communication skills	Animations are animated by coaches in 3d contextual backgrounds on the screen.
A_22 [99]	2020	n.d.	n.d.	n.d.	Experiment	Qualitative	Mobile devices	cognitive skills	The system was built without the involvement of the autism specialist or caregivers

**Figure 14:**
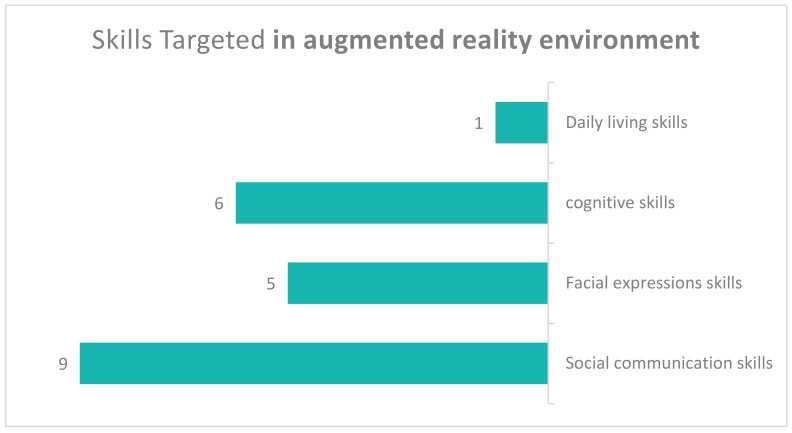
Skills Targeted in augmented reality environment.

- The devices used in AR systems for the rehabilitation of ASD patients were limited. Two studies used smart glasses; six studies used computers (the most common device used) in rehabilitation centres and 12 studies used mobile devices (smartphones, tablets and laptops). Three studies that used smartphones were obtained and another three used a tablet device. We found only one study that used a head-mounted device; one study did not mention the type of device used.
- Figure 14 shows the various skills targeted in the related studies; these can be classified as follows. The largest number of studies (eight) targeted social communication skills, e.g., the effectiveness of asking for help and expressing abilities. Five studies involved facial expression skills and focused on six expressions (anger, fear, disgust, happiness, sadness and surprise). Six studies included cognitive skills, e.g., academic skills (arithmetic, geography, biology, numbers and letters), as well as attention skills, mental representations, focus skills and the recognition of objects. Daily living skills were represented in only one study. One study did not reveal specifically addressed skills.

### 4.2. Serious Games with Autism Spectrum Disorder

Table 9 presents a summary of studies related to the field of serious games. It is presented in terms of year of publication, study type, country, participants, evaluation time, intervention, research strategy, methodology, device type, targeted skills, activity, modality, interaction style, environment and limitations.

**Figure 15:**
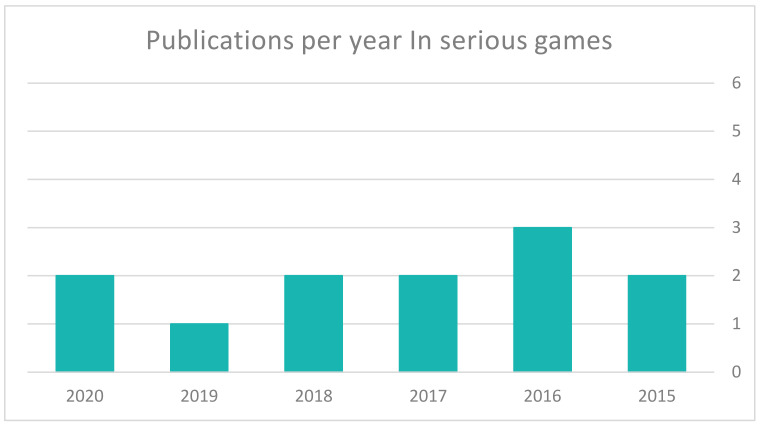
Publications per year in serious games.

Figure 15 shows the number of studies published between 2015 and 2020. Only a small number was published during the past five years; the highest number was in 2016 (at a rate of three studies) and in 2019, this decreased to one study. We expect that this had been due to a lack of game designers interested in the field of ASD rehabilitation. 

The percentage of conference publications (67%) was larger compared with journal articles (33%). Regarding the number of studies per country, six countries (Canada, the United Kingdom, Egypt, the Philippines and Serbia) each published one study. There were six studies for which the country of publication was not noted.

**Table 9:** The related studies of serious games with ASD.

A_ID	Year	Country	Evaluation Time	Research Strategy	Methodology	Device Type	Targeted Skills	Activity	Modality	Interaction Style	Environment	Limitations
S_1 [100]	2020	n.d.	n.d.	case study	Quantity	n.d.	cognitive Skills	Mental	Visual, Auditory	Tangible interfaces	2D	Interpreting and describing all possible reactions in a child ASD is difficult
S_2 [101]	2017	Canada	2 days	Experiment	Qualitative	computer	Social and communication skills	Mental	Visual, Auditory, Haptic	Tangible interfaces	2D	The complexity of the proposed system building tools and needs news to operate. The experiment was only performed on males
S_3 [102]	2018	UK	n.d.	Experiment	Qualitative	laptop	cognitive Skills	Physical exertion, Mental	Visual, Auditory	Kinect and mouse	2D, multi-player games	Cessation and absence of some participants from the experiment.
S_4 [103]	2015	Germany	n.d.	Experiment	Qualitative	Mobile devices	Facial expressions Skills	Mental	Visual, Auditory	Tangible interfaces	2D	There are no motivational phrases when he finishes the stages to encourage the child
S_5 [105]	2020	Egypt	n.d.	Experiment	Qualitative	computer	cognitive Skills	Mental	Visual, Auditory	Keyboarded	2D/3D	The difficulty is configurable to make it more suitable for every child.
S_6 [123]	2016	n.d.	n.d.	Experiment	Qualitative	mobile device	Facial expressions Skills	Mental	Visual, Auditory	Tangible interfaces	2D	It depends on the presence of an official dealing with the program
S_7 [106]	2016	Philippines	n.d.	Experiment	Qualitative	mobile device	cognitive Skills	Mental	Visual, Auditory	Tangible interfaces	n.d.	Lacks the side of interaction and attraction and does not benefit from the standards of serious games
S_8 [107]	2019	n.d.	n.d.	Experiment	Qualitative	Tablets	Social and communication skills	Mental	Visual, Auditory	Tangible interfaces	2D	Limiting the time in the fourth, fifth, and sixth stages may cause the child to become distracted.
S_9 [108]	2017	n.d.	n.d.	Experiment	Qualitative	computer	cognitive Skills	Mental	Visual, Auditory	keyboard and a mouse	3D	Control of the keyboard is difficult for children because there are many commands (such as space to jump, mouse to move the camera, etc.)
S_10 [109]	2016	Serbia	2 weeks	Experiment	Qualitative	computer	Motor and cognitive Skills	Physical exertion, Mental	Visual, Auditory	Kinect	2D	There are no specific periods in the game that the child loses focus
S_11 [110]	2018	n.d.	n.d.	Experiment	Qualitative	computer	cognitive Skills	Mental	Visual, Auditory	n.d.	2D	The serious game concept has not been used in an interestingly and attractively way to the target age group
S_12 [111]	2015	n.d.	n.d.	case study	Qualitative	mobile device	Emotional Skills	Mental	Visual, Auditory	Tangible interfaces	AR	The number of attempts to reach the correct answer is open and may affect the results of the child’s evaluation

**Figure 16:**
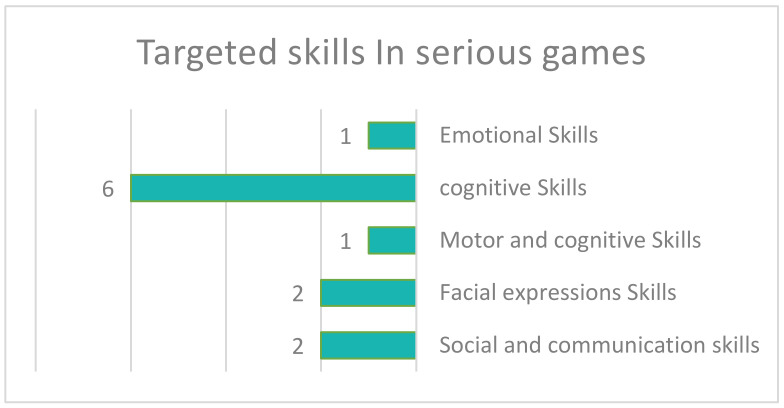
Targeted skills in serious games.

Devices used in serious games for ASD rehabilitation were small in number; five studies included computer use and six included mobile devices (smartphones, tablets and laptops). One study using a tablet device and one employing a laptop were obtained. Figure 16, shows the variety of skills targeted in related serious games studies. The largest number of studies targeted cognitive skills (six), which included skills such as understanding, academic skills, financial concept skills and computational thinking skills. 

This was followed by two studies that involved facial expression skills; two additional studies included social and communication skills. Only one study included emotional skills, motor and cognitive skills.

We previously noted that the activity criterion included three types, i.e., mental, physical and physiological activity. However, physiological activity was not included in the current paper because it targets and treats autistic individuals with impaired movement, as well as visual and auditory sensitivity. All 12 related studies targeted mental activities, in contrast to physical activities, which were implicit in two studies with mental activities; no study focused only on physical activities.

**Figure 17:**
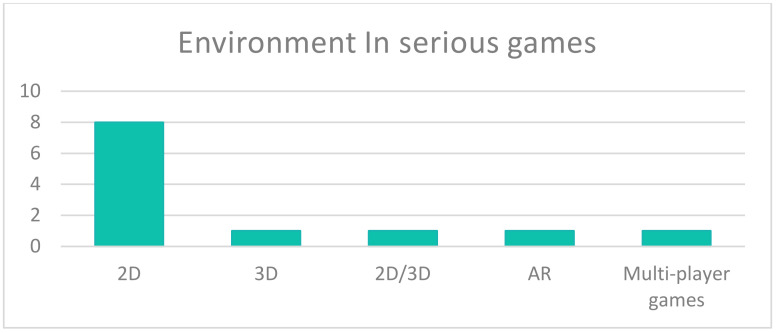
Environment in serious games.

Studies related to serious games included three styles: tangible interfaces, keyboard and/or mouse and the Microsoft Kinect system. Tangible interfaces were used at a rate of 58% and included physical or digital interfaces. Representing a traditional interaction style, a keyboard and/or mouse was used in up to 25% of studies. A relatively new style involved using the Kinect (17%). Figure 17 shows an unexpected consequence of the environments used in serious game studies. Despite the variety of environments in serious games, they were limited to 2D aspects in eight studies. Conversely, 3D, 2D/3D, AR and multi-player games were each represented by one study (excluding studies that included VR).

### 4.3. Applying the Picture Exchange Communication System with Autism Spectrum Disorder

Table 10 summarizes the results of the study on the application of PECS in terms of publication year, type of study, country, participants, and the number of phases applied, evaluation time, device type, intervention, search strategy, methodology and limitations. Only a few studies on the applications of PECS were published (nine in total); the highest value was found in 2018 (four) and in 2019 this decreased to two studies. 

Unlike the fields of AR and serious games, 67% of related studies were published in journals, while conference papers represented 33%. We note that the percentage of studies published in journals was almost twice that of conferences papers.

The PECS studies included three countries, i.e., Taiwan (one study), Indonesia (two) and South America (one). Five studies did not denote a country of origin.

**Figure 18:**
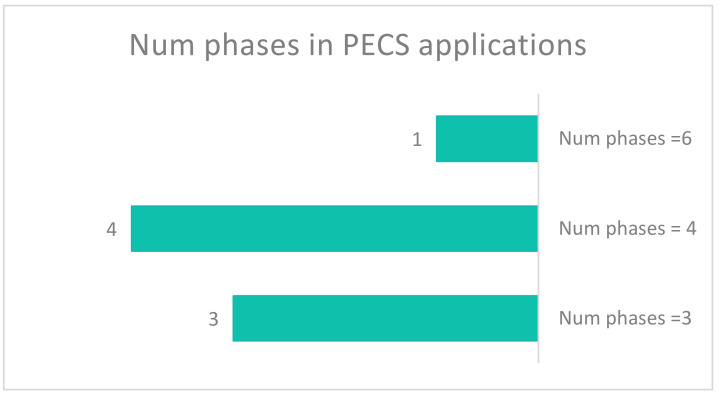
Num phases in PECS applications.

When considering studies related to the number of applied PECS phases, we found that they were limited to the first four phases (One study exception). Figure 18 shows that four studies applied four phases and three studies used three phases. Only one study used all six phases and another only applied the concept of PECS. 

**Table 10:** The related studies of PECS applications with ASD.

A_ID	Year	Type	Country	Participants	Num_Phases	Evaluation Time	Device Type	Intervention	Research Strategy	Methodology	Limitations
P_1 [112]	2015	Journal	Taiwan	11	3	four-week	Tablets	Child-computer interaction	Experiment	Qualitative	The study was based on the concept of PECS, not the application of stages
P_2 [113]	2016	Journal	n.d.	n.d.	3	n.d.	Mobile devices	Augmented reality	quasi-experimental	Qualitative	Conducted on children between the ages of 6 and 11 years. Is long-term use of mobile phones leads to further isolate the child? The evaluation time is short.
P_3 [114]	2018	Journal	n.d.	4	6	n.d.	Mobile devices	Augmented reality	Case study	Qualitative	Using virtual reality instead of augmented reality as the interfaces illustrate this.
P_4 [115]	2018	Journal	Indonesia	12	4	n.d.	smart phones	Augmented reality	experimental	qualitative	Use the traditional PIECE image. The time for an experiment is not clear.
P_5 [116]	2018	Journal	Indonesia	n.d.	4	n.d.	n.d.	n.d.	quasi-experimental	Quantitative	Lacks expressions of encouragement and attraction to the child
P_6 [117]	2019	Conference	South America	9	3	n.d.	Mobile devices	TEACCH Methodology And Autisdata software	experimental	qualitative	I lack attraction and encouragement in the app
P_7 [118]	2018	Journal	n.d.	n.d.	4	n.d.	Mobile devices	n.d.	experimental	Qualitative	The pull-out process can be complicated for a child The images used are not interactive
P_8 [119]	2019	Conference	n.d.	4	4	n.d.	Mobile devices	Goal-Directed Design (GDD) method	experimental	Qualitative	The time is long in long sentences, which causes boredom for children
P_9 [120]	2017	Conference	n.d.	n.d.	n.d.	n.d.	Mobile devices	Aided Learning (CAL), and Human-Computer Interaction (HCI), Mobile Instant Messaging (MIM)	experimental	Qualitative	Only the PECS concept was used and not all stages were implemented

The reason for the most studies being limited to the first four phases was because phases five and six of PECS require people with ASD to answer a set of questions and talk about their feelings; this is not provided for in PECS’s applications.

We found PECS applications to be limited to mobile devices (100%). One study focused on the use of smartphones and one other on using a tablet; six studies all included mobile devices. One study made no mention of the device type.

Among the important results in the techniques and approaches for delivering intervention through the applications of PECS, we found that AR had been used in three studies. The remaining studies were more diversified and included many different technologies and approaches, such as child–computer interaction, the Treatment and Education of Autistic and Related Communication Handicapped Children methodology (TEACCH ^®^) and Autisdata software, the goal-directed design method, aided learning, human–computer interaction and mobile instant messaging.

## 5. Discussion

The main findings related to each RQ and SRQ of the current study is briefly described below.

- MRQ1: What are the effects of applying developed AR to developing the communication and social skills of ASD children?

As previously noted, the studies that targeted social and communication skills included eight in all [A_2], [A_3], [A_4], [A_7], [A_10], [A_16], [A_18] and [A_21]. All results indicated that the use of AR technology had a positive effect on the progress of speech and language therapy in particular, as well as the social skills and communication in general for people with ASD. Moreover, the experience of AR technology was not limited to its impact on social skills and communication; other positive effects such as increased interest in the education process through participation, enjoyment and motivation were also indicated. The educational processes for people with ASD require long-term continuity; therefore, AR technology provides a very enjoyable and stimulating learning environment with a wide variety of sources for learning activities, which can help to avoid students becoming bored quickly. In addition, AR technology was able to deepen an understanding of the relationships between social situations and the different behaviours of individuals with ASD; in this context, it was observed that AR enhanced their social cues. Moreover, through exercises, role-playing games and interactive games using augmented reality, social skills were significantly affected. 

Despite its positive effects, however, there are limitations to the use of AR in the rehabilitation of people with ASD. Scanning signs or objects using camera capturing are not designed in a manner that considers the capabilities of people with ASD. These signs and objects must be in a specific position, have good lighting and must be at a reasonable distance. For AR characters, these were represented by three types in the reviewed studies, i.e., cartoons, avatars and emojis. However, in most studies reviewed in this current research, the gestures and facial expressions of the characters were not considered. Only one study created an avatar; in this case, the faces of the participants were captured by taking several photos at different angles ([A_9]); however, this will be a complicated process if the number of participants is large.

- MRQ2: What is the effect of applying the standards of the serious game developed for children of ASD?

Through their findings, studies relating to the efficient use of serious gaming standards in many learning practices enable their implicit understanding. These findings indicate a positive effect in terms of enhancing the motor skills and interactive functions of people with ASD, particularly children. The results also showed the effectiveness of serious games in terms of improving the language skills of children before and after using the game. Moreover, more interactive games motivated children to work towards common goals. Importantly, serious gaming standards were able to enhance the visual support factor, which must be taken into consideration when working on the rehabilitation of people with ASD. However, studies related to this context were limited to implementing traditional standards and these were not properly applied. Only one study among all those described, employed the AR environment ([S_12]), despite its positive impact on educational activities. Due to the low level of diverse environments in serious games studies, a lack of standard interaction patterns emerged because the environments were limited to the traditional mouse and keyboard interaction methods, as well as tangible interfaces.

- MRQ3: How effective are the developed PECS applications for ASD children?

The effect of PECS applications was effective and positive in all respects. Studies showed that using the benefits of PECS applications effectively improved communication among ASD individuals with communication problems (verbal/social communication). The reasons for improvement included reducing the burden of carrying many traditional cards to make it easier to manage them; PECS applications apply a large range of symbols and images alongside the feature of adding sound effects to teach pronunciation and motivate people with ASD to learn. Moreover, PECS applications alleviated the difficulties faced by caregivers, such as reducing verbal repetition and accessing cards faster, thereby overcoming spatial and time constraints and enabling ASD progress monitoring without indirectly interfering at any time and any place after the completion of the session. Despite the many advantages we have mentioned, the related studies were small in number and several presented limitations. The applications lacked interaction and verbal communication (applied in phases five and six of PECS), providing one study ([P_3]), It is suggested to implement phases 5 and 6 in a cooperative manner between two players, through simple comments, asking for help and giving opinions while playing. Moreover, studies lacked participation and interaction with the caregivers, parents or friends of those with ASD; involving these groups could potentially increase the effectiveness of treatment using PECS’s applications, particularly in advanced stages of training. Most of the studies that addressed the need for the mouth movement feature which focus on clarifying how to pronounce the names of things and the mouth movement while the word is pronounced.

- MRQ4: Can the application of PECS be developed with AR technology?

In the [P_2], [P_3] and [P_4] studies, it emerged that it is possible to design PECS applications using AR technology. The results also indicated an improvement in the ability of verbal communication, but more studies are needed over longer periods. Another major limitation is that the PECS approach has not been effectively integrated with AR technology, as AR was included only in some stages.

(1) SRQ1: Can developing AR serious games attract ASD children?

As we previously noted, one study included in this review was related to serious games that used an AR environment ([S_12]). This study aimed to motivate children with ASD to acquire emotional recognition skills by attracting their attention and stimulating them with AR technology. What distinguishes this study is that its approach represented fun and interesting ways for people with ASD, where the GameBook was used to represent and interact the events of the story using AR technology. In contrast, also the study is a prototype that has not been evaluated for the reliability of serious games using AR technology.

(2) SRQ2: Can all phases of PECS be applied in an AR application

Based on studies [P_3] and [S_8], it seems possible to create a PECS application that supports all six phases of this approach. Studies demonstrated positive results for developing communication and interaction skills through the application of PECS. However, These two studies is insufficient for demonstrating the effectiveness of supporting all phases of the PECS approach in AR application, as further testing involving a larger group of participants is required. Moreover, this study employed AR technology and, as such, are prone to the same limitations that we mentioned.

(3) SRQ3: How long can ASD children continue to be attracted to PECS’s applications?

Maintaining the attention of individuals with ASD during treatment using the PECS approach is a primary obstacle, as treatment sessions for as long as five years may be needed to gain and retain minimum verbal skills. Therefore, attracting the attention of people with ASD for the longest possible period is required. None of the studies included herein could conduct PECS evaluation sessions for long periods; the longest time reached by related studies was four weeks ([P_1]). Accordingly, many more studies that evaluate the attractiveness of using PECS applications and that measure the maximum possible period for maintaining the attention of ASD individuals when using PECS applications are needed. The attraction factor mainly depends on the technologies that are incorporated in PECS applications.

(4) SRQ4: How much can we benefit from previously reviewed papers that target ASD children?

Through an in-depth analysis of all related studies, many aspects emerged that present several areas of potential development for future studies. These aspects are described in the following points.

- Participants: Based on the inclusion criteria for the current survey, all studies involving individuals with ASD that present with one specific symptom (mild, moderate, or severe) and no movement or cognitive impairments, special needs, or visual and auditory sensitivity were reviewed. Participants in the studies related to this survey included children and adolescents with ASD. This a group of ASD did not show symptoms of autism in strangers; but, the situation becomes embarrassing in dealing with them despite their high ability to learn, so they are not treated equally with other groups in which the symptoms of autism are a phenomenon. The different personality traits of people with ASD called for different techniques and interventions to be implemented in their rehabilitation, as most techniques that rely on sound or visual effects may negatively affect people with ASD who have auditory/visual sensitivity. Two included studies addressed the difference in personality traits of people with ASD during the development of the proposed learning systems ([P_3] and [S_4]). By allowing the systems to have flexible configurations to suit the needs of all those with ASD, such as choosing the gender, application color, and volume of sounds in the application etc. In terms of the number of participants, this ranged in most studies from one-to-four participants. These small samples may have been due to the difficulty of dealing with large numbers of individuals with ASD, based on the emotions they may experience during an intervention. Furthermore, each patient needs to be treated independently by a person they are familiar with.
- Device type: Different opinions were observed in the studies regarding the type of device used in the rehabilitation of people with ASD. In 11 of the related studies ([A_4], [A_6], [A_7], [A_9], [A_18], [A_21], [S_2], [S_5], [S_9], [S_10] and [S_11]) the use of traditional devices (computers) was beneficial in terms of the variety of devices that could be linked to it, e.g., adding a motion sensor or large display screens; however, it was restricted in that they could only be used in rehabilitation centres. Additionally, many of these devices were expensive and it may also be confusing for people with ASD to interact with more than one device at the same time. Furthermore, advanced technology devices require an expert to operate and manage them. The use of smart glasses presented potential benefits in two studies ([A_13] and [A_16]). The use of smart glasses was found to be less distracting and less demanding in terms of cognitive workload. This was because smart glasses do not require the use of hands, which is beneficial in terms of not distracting the user and allowing them to only focus their visual attention during the learning process. However, the side- effects of using smart glasses for a long time for people with ASD remain unknown. Moreover, smart glasses are not able to address the visual problems of ASD individuals.

Significant support for the use of mobile devices (smartphones, tablets and laptops) was evident. Smartphones were included in 17 related studies ([A_2], [A_5], [A_15], [A_19], [A_20], [A_22], [S_3], [S_4], [S_6], [S_7], [S_12], [P_2], [P_3], [P_6], [P_7], [P_8] and [P_9]). Four related studies made use of laptops ([A_10], [A_12], [A_14] and [P_4]) and tablets were employed in five related studies ([A_1], [A_3], [A_17], [S_8] and [P_1]). These devices present multiple advantages, such as their portability, the ability to use them at anytime and anywhere, as well as their low cost compared with more traditional devices, which are limited in certain places and times. Moreover, people with ASD exhibited positive reactions when using smartphones and tablets. Despite promising results, however, some studies also expressed concerns about the use of smartphones and tablets, as prolonged and unregulated use can lead to isolation among ASD individuals and reduce their awareness of the surrounding (social and physical) environment; this is particularly the case for ASD individuals who primarily experience a lack of social communication skills. Selected studies showed that smartphones increased stress and pain due to remaining in the same position for an extended time.

Head-mounted devices were used in one study ([A_8]). These represent close-up displays that provide more control for those with ASD in terms of what they can observe, unlike the screens of mobile devices, which have a limited field of view. In addition, head-mounted devices enhance a sense of fun and can present a realistic AR experience. The disadvantage of head-mounted devices appears to be their need for operating in a low-light environment.

- Culture: East Asian culture featured predominantly in the reviewed studies. Interestingly, no established studies were found in an Arabic culture setting. One study derived from Qatar ([A_5]) and another from Egypt ([S_5]); however, these studies depended on having knowledge of the English language or translating it only into Arabic.
- Interventions: By combining the advantages of AR studies, serious games studies and PECS application studies, regardless of the characteristics of the participants, a diversified series of research can be collected that addresses current research problems in correlated studies.

We found one serious games study that employed an AR environment ([S_12]). The study aimed to create a game that would attract children’s attention to develop social and communication skills. This approach used the software Gamebook, which depends on a graphic interface, character interactions, voice and text narration and AR technology by using a children’s book. The AR in Gamebook provides an experience within a safe and attractive environment and can enhance a child’s attention and imagination without diminishing it. Moreover, through the game’s website, the system provides a feature that is able to monitor the results of children without direct intervention, thus supporting the child’s independence. All of these features were included in this work without financial costs or the use of traditional peripheral devices such as a keyboard and mouse.

Conversely, one serious games study also targeted social and communication skills but did so using the PECS method ([S_8]). The game in this study included 15 levels, which were divided as follows. (1) Levels that achieved differentiation and recognition skills through the use of the first, second and third stages of the PECS approach. (2) Levels that achieved the skills of building sentences and communicating with others through the use of the fourth stage of the PECS approach. (3) Levels (14 and 15), in which progress in communication skills was achieved by asking for help and making comments while playing with other players, and which followed a cooperative approach that achieved the values of participation and cooperation between players.

We also found three studies that combined AR with a PECS ([P_2], [P_3] and [P_4]) approach. In [P_2], the first three phases of the PECS approach were implemented alongside an AR technology intervention. Augmented reality technology displayed an animal class as 3D objects in a scene, thereby enhancing the attraction factor for ASD individuals. In the stage that was implemented, the goal was to achieve object recognition skills by clicking on the icon for each animal. The animal that was clicked display for viewing in a 3D image. In [P_3], the 3D objects were expanded because the application was designed with seven menus, each representing a stage of the PECS system; a final list was also presented to exit the game. The first stage comprised five categories, i.e., the classes, a dining room menu, a bathroom menu, a bedroom menu and a playground menu. In each category, all the components of the selected category were displayed. At the end of each stage, a short test was presented to assess ASD children. In [P_4], the first four phases of the PECS approach were used. Here, a conventional PECS card was scanned by smartphone camera and each card was represented by 3D objects. In the related studies, AR was used with PECS applications in a traditional manner, i.e., the representation of objects in 3D form only, without interaction or having a logical sequence of events. We believe that by leveraging the standards of serious gaming and the benefits of an AR environment to develop PECS applications, the problems that emerged in the reviewed studies could be avoided. None of the included studies adopted such an approach.

- Independence: A relationship was observed between the type of devices used in studies and support for autonomy in working on the system without the presence of the device operator for the duration of the treatment session. In studies that relied on using a computer, an expert or care provider was required to operate them or to assist in using peripheral devices. Therefore, mobile devices support independence via their ease of use. Despite this, few studies were directly concerned with independence ([A_6], [A_9], [A_22], [S_1], [S_3], [S_4], [S_8], [S_11], [P_3] and [P_6]).
- Monitoring progress: Enabling the independence of people with ASD and monitoring their progress presents some obstacles. People with ASD cannot be left unattended until the provided treatment has been used and recorded; concurrently, stimulating the independence of those with ASD should also be a focus. Monitoring progress should be an independent process that ensures that treatment is not directly interfered with. A group of studies suggested independent systems that help caregivers and parents monitor progress, either through websites ([S_7] and [S_10]) or by including them in the application ([A_22] and [P_5]). One study was entirely based on assessing the comprehension score of a child with ASD to provide accurate educational assistance ([S_1]). This approach will help caregivers to accurately identify concepts that the child did not fully understand. Conversely, all studies manually implemented progress monitoring, which can be considered inaccurate compared with automatic progress monitoring.
- Cooperation: Selected studies ([A_15], [S_3] and [S_8]) indicated that games that specifically reinforce cooperative behaviours can help to develop the social behaviours of ASD individuals because they enhance an understanding of others, use simple interactive dialogue (such as asking for help) and make use of eye contact. However, these studies explained that some ASD individuals may more frequently engage in collaborative activities only to affect blame and to complain. In some instances, reactions made by ASD individuals may be unexpected in the event of mistakes on behalf of one of the parties involved.

## 6. Ongoing Issues and Future Research Directions

In this section, we provide a summary of ongoing issues and possible future directions for research based on the reviewed studies.

### 6.1. Ongoing Issues

Two types of ongoing issues (general and private) emerged from the included studies. The general issues refer to common issues in all of the reviewed studies, regardless of their particular field. Private issues are those that are specific to one area among those discussed herein.

#### 6.1.1. General Ongoing Issues

- Several participants

The number of children with ASD who participated in all of the reviewed studies was small, ranging in most (20) studies from one-to-four participants. Interestingly, there are few studies that recruited a large number of participants, as the number of study participants was [A_19] reach to 200 participants over a period time of up to 3 years in AR studies. Hence, the included studies do not represent a strong body of evidence as the findings have not been validated by more systematic evaluations.

- Duration of the experiment

Behavioural therapies for people with ASD require long-term skills training. These issues are among the most prominent. In most of the reviewed studies that lasted up to four weeks, it is important to note that the number of sessions had not been counted because there was no clarification on the required sufficient period for acquiring skills. As already stated, one study ([A_19]) spanned a period of three years. We believe that the reason for the short duration of the experiment in this particular study can be ascribed to the fact that many of the studies had been conducted for specific conferences; accordingly, the duration of the experiment was adapted based on the timing of these conferences.

- Completing the experiment

Completing an experiment can be a difficult task for people with ASD. We observed in most of the studies the withdrawal of one or two participants from the experiment for several reasons. One of these reasons related to experimental difficulties, that is, either because of the complexity of the system or because of the selection of participants whose characteristics did not match the experience. Other causes were related to the mood and physical state of people with ASD (aspects such as tension, fear and hunger must be considered). Furthermore, in some studies, people with ASD did not interact with researchers because they did not feel comfortable interacting with strangers.

- Duration of use of mobile devices

Despite the reported positive results and stimulus interaction resulting from ASD individuals using mobile devices, there is a worrying trend related to the use of these devices. Evidence in studies indicates that the use of mobile devices can cause feelings of isolation for people with ASD, which may reduce their awareness of social and physical environments, particularly for ASD individuals with reduced communication and social skills. As such, prolonged use of these devices during the treatment period may contribute to a deterioration of these skills.

- Culture

As previously noted, most of the reviewed studies were based in East Asia, indicating a lack of other additional cultural approaches in related studies. The bulk of studies were written in the English language or propose a studies based to the translation of the English language without carrying the original culture of the country of origin for the study. The images used in these studies represent selected cultures and may thus reduce the effectiveness of the social communication process. None of the included studies focused on studying the effect of culture and AR applications on the rehabilitation of people with ASD.

#### 6.1.2. Private Ongoing Issues

- Augmented Reality

The main problem in studies related to AR environments is that these environments involve only a display of objects with/without cards. As such, they lack a connection to any logical sequence of events. Moreover, although applications that rely on AR environments are being developed, few of them consider the needs of people with ASD in their communication with caregivers or with other ASD individuals. Moreover, a technical issue related to displaying AR objects currently appears unclear; the cause may be due to shaking of the camera device, particularly shaking caused by ASD individuals. Another reason may be attributed to lighting. In addition, in the studies included herein, these applications lacked response speed or a sense of real interaction with ASD sufferers.

- Serious Games

One of the most prominent issues regarding serious games was the lack of diversity in the applied standards; furthermore, these standards were not properly employed. Due to the lack of diverse environments in the studies focused on serious games, an absence of standards for the interaction type was evident because this was limited to traditional methods. Furthermore, as was the case for the development of AR applications, which are limited to being created by software developers, most studies related to serious games often developed applications that lacked a sense of fun. A reason for this may be because the games are designed for sponsor providers or researchers who did not consider using creative game designers who are better equipped to create applications that ensure a focus on fun and engaging users.

- Picture Exchange Communication System (PECS)

The lack of applications using PECS have makes it a poorly researched topic with several obstacles. Most PECS applications were applied in a traditional manner that simulated the use of a manual card. These were primarily 2D digital displays with added sounds; as such, they lacked visual and sensory interaction. Moreover, based on this design, the application made the process of moving between images difficult, either because of the size of the image, which was often small or, alternatively, because of the process of dragging images to form sentences. As we previously noted, most of the PECS applications were limited to the first four stages, creating issues linked to reduced social interaction, which the final two stages of PECS can provide.

### 6.2. Future Research Directions

The information presented in the ‘Discussion’ and ‘Open Issues’ sections can assist in predicting future trends that may contribute to additional studies linked to developing the field of rehabilitation for people with ASD.

Participants

By reviewing the number of participants and the personal characteristics that must be considered in studies related to the rehabilitation of people with ASD, future studies may opt to include their number of participants. Autism centres can be contacted to carry out a systematic evaluation of up to 12 participants, both male and female, with no other diagnoses except for ASD. The age group ranging from three to 12 years of age can also be included. This age group is able to rapidly acquire communication and social skills but is the least focused on by ASD research.

Device type

We support the use of mobile devices for several reasons including their attractiveness to people with ASD. Moreover, they can be used almost anywhere without significant financial costs and supports the pattern of repetition required by behavioural therapies for people with ASD. It is possible to avoid the isolation that accompanies frequent and prolonged use of mobile phones in future studies by imposing specific periods of use, e.g., during treatment sessions in clinical centres. This can be applied by adding a feature for caregivers through which to set the time for using the application, from one hour to three as a maximum.

Culture

Studies can be enriched in the future by integrating local culture in the rehabilitation of people with ASD. We aim to create an application that represents Arabic culture through the creation of characters depicted in local dress or engaging in social customs to help ASD individuals acquire and reinforce Arab social behaviours.

Interventions

**Figure 19:**
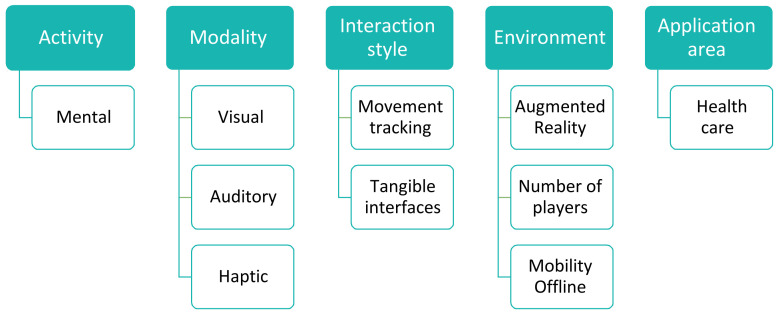
Serious Games Criteria for Future Study.

The incorporation of PECS applications in serious AR environment-based games can provide solutions that ensure that the limitations present in each of the studied areas are improved in future research. An AR environment can provide 3D characters in a simulated real environment to help ASD individuals gain social skills in an attractive and comfortable environment for people with ASD. Moreover, serious games provide a logical sequence of events by following a storytelling approach, which can guarantee aspects such as fun and suspense and implicitly enhance social skills. By carefully diversifying the standards of serious games, educational environments can be created. In future studies, we aim to apply all of the standards shown in Figure 19. In this regard, for the PECS methodology, all six stages can be applied, including a treatment sequence that will provide an implicit aspect in serious games. 

Independence

Using mobile devices can enable people with ASD to be autonomous because they can use the application without assistance. In future studies, this can provide independence through the creation of simple application interfaces, alongside tips that can help guide ASD individuals during the application experience.

Monitoring progress

Relevant studies on monitoring progress can be useful for future research. A progress monitoring feature can be included in the application to allow caregivers to accurately monitor the progress of ASD individuals. A breakdown of each stage completed and the time it took the participant to complete it can be included. Charts can also be added to facilitate the monitoring of participant progress.

Cooperation

Consideration should be given to the problem of how cooperation skills can be enhanced within the target skill framework, particularly when more than one player is involved and the game is conducted over the Internet. Accordingly, in future studies, an AR avatar will be added to represent a parent or friend of the ASD individual. This will be done by adding an image of the individual and having the application create an avatar using said image. People with ASD will then be able to engage in a range of simple conversations and collaborate with the avatar to finish the skill-set target frameworks alongside a familiar character.

## 7. Conclusions

Augmented reality environments have recently gained significant attention in a range of different contexts. This environment can be used efficiently if it is combined with the rehabilitation of people with ASD, e.g., using serious games and PECS applications. In this paper, a rigorous SLR was conducted about these approaches using 172 research papers in three domains published from 2015 to 2020. In the final stage, a total of 43 papers were examined. Augmented reality was represented in 22 studies, serious games studies represented 12 studies and a total of nine PECS application studies were included. We began by detailing the background and relationship between these three areas. We then reviewed existing surveys related to these three areas and found no other surveys related to PECS applications. This is one of the main contributions of this survey. We highlighted the studies related to each field in detail and noted their results. We also discussed the benefits of these studies for the research community and highlighted how future studies can be informed by making use of our review findings. Finally, we provided insights about the remaining issues and future directions for research to improve the application of PECS alongside AR-based serious games.

## Figures and Tables

**Table 4:** The difference between augmented reality and virtual reality environment.

Augmented Reality Environment	Virtual Reality Environment
Provides digital components in a real environment	A completely digital environment
Most of the time, peripheral devices are not required.	Most of the time, many peripheral devices are required (such as controllers, physical devices, etc....)
Requires technical tools to bring together the real and digital environments	Requires technical tools to show digital environmental
It enhances feelings of experiencing in the real world	It enhances feelings of experience in a whole new and safer environment

**Table 5:** Database query and filter actions.

Database	Query	Filters Applied
Science Direct	(“Autism” OR “Autism Spectrum Disorder” OR “ASD”) AND (“Augmented reality App” OR “AR”) AND (“Skills”) AND (“educate” OR “learn”)	✓ Year: 2015–2020 ✓ Article type: Research articles ✓ Subject areas: Computer Science and Engineering ✓ Without “*” ✓ Use fewer Boolean connectors (max 8 per field)
	(“Autism” OR “Autism Spectrum Disorder” OR “ASD”) AND (“Serious games” OR “SG” OR “Serious games App”) AND (“Skills”) AND (“educate” OR “learn”)	
	(“Autism” OR “Autism Spectrum Disorder” OR “ASD”) AND (“PECS” OR “Picture Exchange Communication System” OR “PECS App”) AND (“educate” OR “learn”)	
Springer Link	(“Autism” OR “Autism Spectrum Disorder” OR “ASD”) AND (“Augmented reality” OR “Augmented Reality Therapy” OR “AR” OR “Rehabilitation with augmented reality” OR “ Augmented reality App*”) AND (“*Skills”) AND (“educat*” OR “learn*”)	✓ Year: 2015–2020 ✓ Article type: Articles ✓ Language: English ✓ Discipline: Computer Science
	(“Autism” OR “Autism Spectrum Disorder” OR “ASD”) AND (“Serious games” OR “Applied game” OR “SG” OR “Serious games App*”) AND (“*Skills”) AND (“educat*” OR “learn*”)	
	(“Autism” OR “Autism spectrum disorder” OR “ASD”)AND (“PECS” OR “Picture Exchange Communication System” OR “PECS APP*”) AND (“educat*” OR “learn*” OR “train*” OR “gam*”)	
Semantic Scholar	(“Autism*” OR “ASD”) AND (“Augmented reality” OR “Augmented reality App*”) AND (“*Skills”) AND (“educat*” OR “learn*” OR “gam*”)	✓ Year: 2015–2020 ✓ Article type: Journal Article and Conference ✓ Subject areas: Computer Science and Engineering
	(“Autism*” OR “ASD) AND (“Serious games” OR “SG” OR “Serious games App*”) AND (“*Skills” ) AND (“educat*” OR “learn*” OR “train*”)	
	(“Autism*” OR “ASD”) AND (“PECS” OR “Picture Exchange Communication System”) AND (“App*”) AND (“educat*” OR “learn*” OR “gam*”)	
IEEE Explorer	(“Autism” OR “Autism spectrum disorder” OR “ASD” OR “Asperger syndrome” OR “Rett syndrome” OR “Childhood disintegrative disorder” OR “Pervasive Developmental Disorder–Not Otherwise Specified”) AND (“Augmented reality” OR “Augmented reality therapy” OR “AR” OR “Rehabilitation with augmented reality” OR “Augmented reality App*”) AND (“Social skills” OR “communication skills” OR “*Skills”)AND (“educat*” OR “learn*” OR “train*” OR “gam*” OR “simulat*”)	✓ Year: 2015–2020 ✓ Article type: Journal and Conference
	(“Autism” OR “Autism spectrum disorder” OR “ASD”OR “Asperger syndrome” OR “Rett syndrome” OR “Childhood disintegrative disorder” OR “Pervasive Developmental Disorder–Not Otherwise Specified”) AND (“Serious games” OR “Applied game” OR “SG” OR “serious games App*”) AND (“Social skills” OR “communication skills” OR “*Skills”)AND (“educat*” OR “learn*” OR “train*”OR “simulat*”)	
	(“Autism” OR “Autism spectrum disorder” OR “ASD” OR “Asperger syndrome” OR “Rett syndrome” OR “Childhood disintegrative disorder” OR “Pervasive Developmental Disorder–Not Otherwise Specified”) AND (“PECS” OR “Picture Exchange Communication System” OR “PECS App*”) AND (“educat*” OR “learn*” OR “train*” OR “gam*”)	
Google Scholar	(“Autism*” OR “ASD”) AND (“Augmented reality” OR “Augmented reality App*”) AND (“*Skills”) AND (“educat*” OR “learn*” OR “gam*”)	✓ Year: 2015–2020 ✓ Language: English
	(“Autism” OR “Autism spectrum disorder” OR “ASD” OR “Rett syndrome” OR “Childhood disintegrative disorder” OR “Pervasive Developmental Disorder–Not Otherwise Specified”) AND (“Serious games” OR “Applied game” OR “SG” OR “Mobile serious games”) AND (“Social skills” OR “communication skills” OR “*Skills”)AND (“educat*” OR “learn*” OR “train*”OR “simulat*”)	
	(“Autism*” OR “ASD”) AND (“PECS” OR “Picture Exchange Communication System”) AND (“App*”) AND (“educat*” OR “learn*”OR “gam*”)	

## Data Availability

Not applicable.
